# Integrative single-cell and computational pathology analyses reveal a C11orf24-associated stem-like epithelial population linked to tumor microenvironment remodeling and immune evasion in bladder cancer

**DOI:** 10.1016/j.tranon.2026.103082

**Published:** 2026-10-21

**Authors:** Yingjian Wang, Songxin Zhang, Xi Luo, Zhe Wang, Yongjie Wang, Ningyang Li, Yu Liu, Jingwei Gao, Ranran Zhang, Zhankui Jia, Run Shi, Long Zhang

**Affiliations:** a Department of Urology, The First Affiliated Hospital of Zhengzhou University, No. 1 Jianshe East Road, Zhengzhou, Henan, 450052, China; b Department of Oncology, The First Affiliated Hospital of Nanjing Medical University, Nanjing, China; c Department of Oncology, The First Affiliated Hospital of Zhengzhou University, No. 1 Jianshe East Road, Zhengzhou, Henan, 450052, China; d Proteomics and Cancer Cell Signaling Group, German Cancer Research Center (DKFZ), Heidelberg, Germany; e The First Clinical Medical College of Zhengzhou University, Zhengzhou, China; f Department of Urology, Zhongda Hospital, School of Medicine, Southeast University, Nanjing, 210009, China

**Keywords:** Bladder cancer, Cancer stemness, Single-cell and spatial transcriptomics, C11orf24, Computational pathology

## Abstract

**Background:**

Cancer stem-like cells drive tumor progression and therapeutic resistance, yet the epithelial stemness hierarchy in bladder cancer (BLCA) remains poorly defined. We aimed to characterize stemness-associated epithelial populations and their clinical significance.

**Methods:**

We analyzed single-cell RNA sequencing data from 92,218 cells across 13 treatment-naïve BLCA samples to characterize epithelial stemness and tumor–microenvironment interactions. Spatial transcriptomics and independent bulk cohorts were used for orthogonal and clinical validation. An HStem-associated histopathology-based prognostic model was developed by integrating quantitative image features with deep-learning representations, followed by functional validation of the candidate gene C11orf24.

**Results:**

We identified a highly stem-like epithelial population (HStem) at the apex of the differentiation hierarchy, exhibiting MYC activation, oxidative phosphorylation, and DNA repair enrichment, with preferential presence in advanced-stage tumors. HStem cells were inferred to serve as major communication hubs within the TME, interacting extensively with fibroblasts and endothelial cells via MIF–CD74/CXCR4 and MIF–CD74/CD44 axes. An 86-gene HStem signature enabled development of a robust image-based prognostic model. High-risk tumors showed immune exclusion, immunosuppressive enrichment, T-cell dysfunction, and predicted checkpoint blockade resistance. C11orf24 emerged as a candidate stemness-associated regulator; C11orf24-positive cells displayed enhanced stemness and stromal interactions. Functional experiments demonstrated that C11orf24 perturbation altered clonogenic growth, migration, and invasion in BLCA cells. Pharmacogenomic analyses identified predicted therapeutic vulnerabilities that warrant experimental validation.

**Conclusions:**

We identify an HStem population associated with BLCA progression, microenvironmental remodeling, and immune evasion. Integrating single-cell and spatial transcriptomics with computational pathology, we establish a clinically relevant stemness framework and identify C11orf24 as a potential therapeutic target, offering opportunities for stemness-directed precision oncology.

## Highlights

• HStem defines a stem-like epithelial state at the apex of bladder cancer.
• HStem cells are associated with stromal niches and predicted MIF crosstalk.
• An HStem-based pathology model predicts bladder cancer outcomes.
• High HStem risk marks immune exclusion and predicted checkpoint resistance.
• C11orf24 is a candidate stemness regulator and potential therapeutic target.

## Graphical abstract

**Figure:**
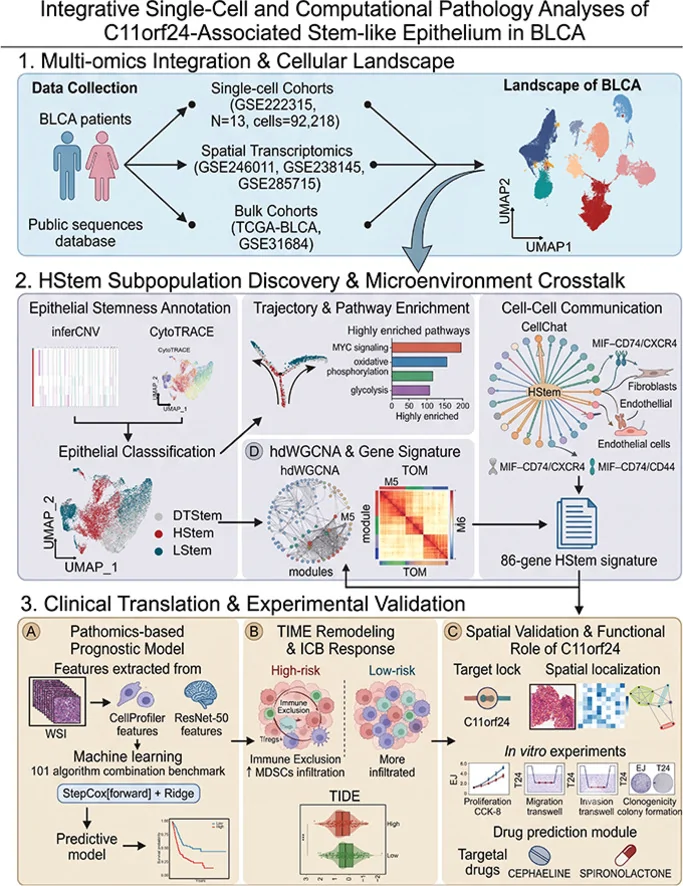

## Introduction

Bladder cancer (BLCA) is among the most common malignancies of the urinary system and remains a major cause of cancer-related morbidity and mortality worldwide[1]. Despite advances in surgical management, systemic chemotherapy, and immune checkpoint blockade (ICB), clinical outcomes for patients with advanced disease remain unsatisfactory owing to frequent recurrence, metastatic dissemination, and therapeutic resistance[2]. Notably, substantial heterogeneity exists both within and between tumors, resulting in divergent clinical behaviors and treatment responses even among patients with similar clinicopathological characteristics. Elucidating the cellular and molecular basis of this heterogeneity is therefore essential for the development of more effective precision oncology strategies in BLCA[3].

Accumulating evidence suggests that cancer stem-like cells (CSCs) play central roles in tumor initiation, progression, metastasis, and treatment resistance[4]. Through their capacities for self-renewal, phenotypic plasticity, and multilineage differentiation, CSCs are thought to sustain tumor evolution and contribute to disease relapse following therapy[5]. In BLCA, stemness-associated transcriptional programs have been linked to aggressive clinicopathological features and unfavorable prognosis[6]. However, the cellular hierarchy underlying malignant epithelial stemness remains incompletely characterized. In particular, it remains unclear whether distinct stem-like epithelial populations exist within BLCA, how they relate to tumor differentiation trajectories, and how they interact with the surrounding tumor microenvironment (TME) to influence disease progression[7].

Recent advances in single-cell RNA sequencing (scRNA-seq) have provided unprecedented opportunities to dissect tumor ecosystems at cellular resolution[8,9]. Single-cell studies have revealed extensive heterogeneity among malignant epithelial cells and identified diverse immune and stromal populations that collectively shape tumor behavior[10]. Nevertheless, most previous investigations have primarily focused on cataloging cellular diversity, whereas the developmental relationships among malignant epithelial subpopulations and the biological mechanisms linking epithelial stemness to microenvironmental remodeling remain poorly understood. Furthermore, although spatial transcriptomics has enabled the investigation of cellular organization within intact tissues, the spatial architecture and ecological interactions of stem-like epithelial cells in BLCA remain largely unexplored[11,12].

In parallel, computational pathology and artificial intelligence have emerged as powerful tools for extracting clinically relevant information from routine histopathological images[13,14]. Increasing evidence suggests that histomorphological patterns can capture underlying molecular states and predict patient outcomes[15]. However, most image-based prognostic models remain largely data-driven and lack mechanistic biological interpretation[16]. Bridging the gap between single-cell-derived biological insights and clinically accessible pathological features may provide an attractive strategy for translating molecular discoveries into practical prognostic and therapeutic applications[17,18].

Here, through the integrative analysis of single-cell transcriptomics, spatial transcriptomics, computational pathology, and functional experiments, we identify a previously unrecognized highly stem-like epithelial population (HStem) that occupies the apex of the malignant epithelial differentiation hierarchy in BLCA[19]. We found that HStem cells exhibited high inferred communication activity within the tumor ecosystem and preferentially interacted with fibroblasts and endothelial cells, while HStem-associated states were linked to immune exclusion and predicted resistance to immunotherapy. Leveraging HStem-associated transcriptional programs, we further develop and validate an image-based prognostic model that enables robust risk stratification from routine histopathological images. Finally, we identify C11orf24 as a candidate regulator associated with the HStem phenotype and experimentally demonstrate its effects on malignant phenotypes in BLCA cells. Together, our findings establish a comprehensive framework linking epithelial stemness, microenvironmental remodeling, pathological imaging features, and clinical outcomes in bladder cancer.

## Material and methods

### Data collection

Single-cell RNA sequencing (scRNA-seq) data for bladder cancer (BLCA) were obtained from the public dataset GSE222315. Spatial transcriptomics data were derived from GSE246011, GSE238145, and GSE285715 for spatial validation. Bulk transcriptomic profiles were obtained from UCSC Xena, including TCGA-BLCA, with the additional validation dataset GSE31684 downloaded from the Gene Expression Omnibus (GEO).

### Single-cell transcriptome analysis and marker gene identification

Raw single-cell transcriptomic profiles were analyzed within the Seurat environment (v4.3.0). Quality-control procedures excluded cells containing fewer than 500 detected transcripts as well as cells exhibiting mitochondrial transcript proportions greater than 15%. Artificial multiplets were subsequently identified and eliminated using DoubletFinder (v2.0.4). To reduce technical variability among samples, data integration was carried out with the Harmony algorithm. Genes displaying the highest expression variability across cells were ranked, and the 2000 most variable features were retained for downstream analyses. Principal component decomposition was then performed, and clustering was generated using the first 50 principal components. Cellular distributions were projected into a two-dimensional space using Uniform Manifold Approximation and Projection (UMAP). Cell identities were assigned according to established lineage-associated marker genes. Cluster-enriched transcripts were determined using the FindAllMarkers function (min.pct = 0.25, logfc.threshold = 0.25, only.pos = TRUE). Statistical inference relied on the Wilcoxon rank-sum approach, and resulting P values were corrected for multiple comparisons using the Benjamini–Hochberg false discovery rate procedure.

### Histopathological image processing and feature extraction

Digital histopathology slides were subdivided into contiguous 512 × 512-pixel regions at 20 × or 40 × magnification. Latent image representations were obtained from each region using a ResNet50 network initialized with ImageNet weights, producing 2048-feature embeddings for downstream analyses.

In parallel, interpretable quantitative histomorphometric attributes were derived using CellProfiler (v4.2.8). These features included measurements of cellular morphology, staining intensity, texture, granularity, and spatial organization. For example, Total_Granularity_8_OrigGray represents a quantitative image-texture descriptor that summarizes intensity variation and spatial organization at a defined granularity scale in the original grayscale image. It was therefore interpreted as a quantitative morphological descriptor rather than a direct molecular biomarker or a surrogate for a specific biological pathway.

Latent image representations were extracted using ResNet50 and consisted of high-dimensional features learned from histopathological image patterns. Unlike CellProfiler-derived descriptors, these ResNet-derived features do not have a one-to-one correspondence with a specific molecular pathway, cell type, or histological structure. They were therefore treated as latent visual representations that capture complex and potentially distributed morphological patterns without assigning a specific biological meaning to individual features.

By integrating deep learning-derived features with CellProfiler-based quantitative descriptors, our framework captures both high-level histopathological patterns and biologically interpretable morphological traits, thereby improving the robustness and interpretability of downstream prognostic modeling.

### Copy-number alteration inference

Genomic copy-number alterations within epithelial populations were estimated using the inferCNV package (v1.24.0). A reference matrix was generated from normal epithelial cells, against which transcriptional profiles of individual epithelial cells were compared. The resulting chromosome-scale alteration landscapes were subsequently utilized to discriminate transformed epithelial populations from their non-transformed counterparts.

### Stemness estimation and classification of malignant epithelial cells

Cellular developmental potential was quantified with CytoTRACE (v0.3.3), a computational framework that predicts differentiation states from transcriptomic complexity. Each cell received a score ranging between 0 and 1, with larger values reflecting a less differentiated and more stem-like phenotype. To minimize batch-related heterogeneity among samples, data integration and correction were performed using ScanoramaCT before score calculation. Tumor epithelial cells were subsequently ordered according to their CytoTRACE scores and categorized into three operational groups. Cells above the 75th percentile were designated as highly stem-like (HStem), whereas cells below the 25th percentile were assigned to the low stem-like (LStem) group. Cells between these two thresholds were classified as differentiation-transition stem-like (DTStem), representing the intermediate portion of the continuous stemness spectrum. The 25th and 75th percentiles were selected as distribution-based operational thresholds to capture the lower and upper extremes of the continuous CytoTRACE distribution while retaining the intermediate population rather than imposing a single arbitrary cutoff around the median. These thresholds were not intended to represent discrete biological boundaries or universally established stemness thresholds. DTStem cells were retained in downstream trajectory and comparative analyses, whereas the principal HStem-associated molecular signature was derived from the biologically contrasting HStem and LStem populations. Accordingly, the HStem-to-LStem relationship was interpreted as a computationally inferred differentiation continuum rather than a deterministic lineage relationship.

### Signaling pathway activity estimation

Intracellular signaling activities were inferred using PROGENy (v1.24.0). Pathway scores were generated through the AUCell-based implementation (run_aucell) together with the curated PROGENy interaction framework. Following recommended practices for single-cell transcriptomic datasets, the highest-ranking 1000 pathway-responsive genes were incorporated into the analysis.

### Biological function and enrichment analyses

Functional annotation was carried out using clusterProfiler (v4.6.2). Over-representation analysis and gene set enrichment analysis were performed against Gene Ontology (GO), Kyoto Encyclopedia of Genes and Genomes (KEGG), and Hallmark collections. Enriched categories meeting a false discovery-adjusted significance threshold of P_adj < 0.05 were retained for interpretation.

### Pseudotemporal reconstruction of cellular dynamics

Developmental trajectories were reconstructed with Monocle2 (v2.26.0). Epithelial subpopulations were assembled into a CellDataSet object using the negative binomial model (expressionFamily = negbinomial.size). Ordering genes were selected according to their expression characteristics and dispersion estimates, after which cells were arranged along a continuous pseudotemporal axis. Low-dimensional embedding was generated through DDRTree (reduction_method = "DDRTree", max_components = 2), and lineage structures were displayed using trajectory plots. Genes exhibiting dynamic behavior throughout pseudotime progression were identified by differentialGeneTest with a significance cutoff of q < 1 × 10−5. These genes were subsequently visualized in heatmap form and clustered according to their temporal expression trends.

### Regulatory network reconstruction

Gene regulatory circuitry was characterized using pySCENIC v0.12.1, a computational framework that integrates transcriptional co-expression patterns with cis-regulatory motif information to identify active regulons. Differentially activated transcription factors across cellular subpopulations were determined using the Wilcoxon rank-sum test. To further quantify the degree of subtype selectivity for each regulon, regulon specificity scores (RSS) were calculated based on Jensen–Shannon divergence implemented in the philentropy package (v0.6.0).

### Intercellular signaling analysis

Communication events among cellular populations were investigated with CellChat (v2.2.0) using single-cell transcriptomic profiles. Ligand–receptor associations were annotated according to the CellChatDB.human resource. The probability of signaling exchange between distinct cell groups was estimated from the expression patterns of interacting molecules, and interactions satisfying a significance criterion of P < 0.05 were retained for subsequent network analyses.

### hdWGCNA

To explore the transcriptional programs characterizing HStem, hdWGCNA was applied to malignant cells. Co‑expression networks were established, and gene modules were detected via topological overlap analysis. Modules showing the strongest association with the HStem phenotype were identified through module‑trait correlation. Genes in these modules were ranked according to their module membership (KME), and the top 100 genes were designated as HStem‑associated hub genes. To identify a robust HStem-associated gene signature, genes from the HStem-related modules identified by hdWGCNA were intersected with differentially expressed genes between HStem and LStem malignant cells. This yielded an 86-gene HStem signature that was used for subsequent analyses. The complete gene list is provided in Supplementary Table S1.

### Construction and validation of the pathomics-based prognostic model

To construct a prognostic model for bladder cancer (BLCA), pathomics features were first screened for their association with the stemness score. Features significantly correlated with the HStem signature score (P < 0.05) were retained for further analysis. Subsequently, univariate Cox regression analysis was performed to evaluate the association of these features with overall survival (OS). A total of 17 pathomics features that were significantly associated with patient survival (P < 0.05) were identified and used for subsequent model development.

A multi-algorithm survival modeling workflow was developed using the Mime platform. This framework integrates several predictive methods, including RSF, Enet, StepCox, CoxBoost, plsRcox, SuperPC, GBM, survivalsvm, ridge, and Lasso. To comprehensively compare predictive performance, 101 candidate workflows generated from different algorithm combinations were examined using 5-fold cross-validation during model development, thereby minimizing overfitting and improving model reliability.

Among all evaluated candidates, the workflow combining forward-selection Cox regression with ridge penalization achieved the highest overall performance. Individual prognostic scores were generated from histopathological image-derived variables, after which patients were assigned to either a favorable- or unfavorable-risk category according to the median score. Model discrimination was quantified using concordance metrics and survival curve analyses. Comparable predictive performance observed in both model-development and evaluation datasets indicated good generalizability of the final signature. The optimal StepCox(forward) + Ridge model, including the selected features and their coefficients, was finalized and locked after model development in the training cohort and was then applied unchanged to the validation cohort. Risk scores for validation-cohort patients were computed using this locked model, and patients were stratified using the same median-based threshold definition. Performance in the validation cohort was evaluated using Kaplan–Meier analysis, time-dependent ROC analysis, concordance metrics, and Cox regression adjusted for clinical variables.

### Spatial transcriptomic data analysis

Spatial transcriptomic datasets generated from ST and Visium platforms were analyzed with Seurat (v4.3.0). Low-quality capture locations expressing fewer than 200 genes, together with genes detected in fewer than three locations, were excluded from further analysis. Variance stabilization and normalization were carried out using SCTransform. Cross-sample integration was achieved through the Seurat workflow incorporating SelectIntegrationFeatures, PrepSCTIntegration, FindIntegrationAnchors, and IntegrateData. Principal component analysis was performed using the top 30 principal components, followed by graph-based clustering. Spatial domains were annotated according to histopathological morphology observed in hematoxylin–eosin sections and cluster-enriched genes. Spatial distributions of clusters and genes were displayed using SpatialDimPlot and SpatialFeaturePlot. To estimate cellular composition within each spatial location, Robust Cell Type Decomposition (RCTD) was applied in full-mode, leveraging single-cell transcriptomic references. Differential markers for individual cell populations were identified with FindAllMarkers using positive log2 fold-change criteria. Interactions among spatially distributed cell populations were subsequently evaluated using MISTy (mistyR v1.16.0).

### Characterization of immune features and prediction of therapeutic benefit

Immune-related characteristics were assessed with the IOBR framework, which was used to estimate both leukocyte infiltration levels and immune signaling activities. In parallel, single-sample gene set enrichment analysis was conducted to quantify predefined immunological programs. The likelihood of response to immune checkpoint inhibition was estimated using the Tumor Immune Dysfunction and Exclusion (TIDE) algorithm. This method models mechanisms associated with T-cell impairment and restricted immune infiltration. Elevated TIDE values were interpreted as evidence of stronger immune escape and reduced sensitivity to checkpoint-directed therapies.

### Prognostic evaluation and clinicopathological association analyses

Patient survival outcomes were investigated using the survival package in R. Associations between variables and overall survival were quantified through proportional hazards modeling, yielding hazard ratios together with corresponding 95% confidence intervals. Survival probabilities were estimated by the Kaplan–Meier method, whereas statistical differences between groups were examined using log-rank testing. Differences in immunotherapy response frequencies were evaluated with Pearson's chi-square test.

### RNA extraction and quantitative PCR

Total RNA was isolated from cultured cells using the SPARKeasy Improved Tissue/Cell RNA Kit (AC0202-A; Shandong Sparkjade Biotechnology Co., Ltd.). Complementary DNA was generated with the NovoScript® Plus All-in-One First-Strand cDNA Synthesis SuperMix (E047–​01A; Novoprotein, Shanghai, China). Quantitative PCR experiments were carried out using NovoStart® SYBR qPCR SuperMix Plus (E096–​01A; Novoprotein) on a QuantStudio™ 3 Real-Time PCR platform (Thermo Fisher Scientific). Relative transcript abundance was normalized to β-actin expression. Oligonucleotide primer information is provided in Supplementary Table S2.

### Plasmid construction and shRNA reagents

Expression vectors encoding Flag-tagged C11orf24 and shRNA constructs targeting C11orf24 were commercially generated by Genechem (Shanghai, China). Lentiviral packaging vectors pMD2.G and psPAX2 were obtained from the same supplier. Additional short hairpin RNAs were purchased from Hanbio (Shanghai, China), whereas shRNA expression constructs were acquired from RiboBio (Guangzhou, China). Target sequences are summarized in Supplementary Table S3.

### Clonogenic growth assay

To determine long-term proliferative potential, EJ and T24 cells carrying C11orf24 overexpression or silencing constructs were plated into six-well culture dishes at approximately 800 cells per well. Following incubation for nearly two weeks, colonies were fixed with paraformaldehyde and visualized using crystal violet staining. Aggregates consisting of at least 50 cells were recorded microscopically and used for quantitative comparison.

### Wound-repair migration assay

Cells were cultured in six-well plates until reaching near-complete confluence. A uniform gap was introduced using a sterile pipette tip, and detached cells were removed by phosphate-buffered saline rinses. Cultures were then maintained under reduced-serum conditions. Images were captured immediately after scratching and again after 24 h. Migration capacity was estimated by calculating the percentage reduction in wound area with ImageJ software.

### Transwell-based motility and invasion experiments

Cell migratory and invasive behavior was examined using Transwell inserts containing membranes with 8-μm pores. For migration measurements, serum-starved cells were added to the upper compartment, while complete medium supplemented with fetal bovine serum served as a chemoattractant in the lower compartment. For invasion experiments, inserts were coated with Matrigel before seeding. After the designated incubation period, cells remaining on the upper surface were removed. Cells that traversed the membrane were fixed, stained, imaged, and counted in randomly selected microscopic regions.

### Western blot analysis

Cells with C11orf24 overexpression or knockdown were lysed using RIPA buffer supplemented with protease and phosphatase inhibitors. Equal amounts of protein were separated by SDS–PAGE and transferred onto PVDF membranes. Membranes were blocked and incubated with primary antibodies against C11orf24, SOX2, ALDH1A1, CD44, and an appropriate loading-control protein, followed by incubation with HRP-conjugated secondary antibodies. Protein signals were detected using an enhanced chemiluminescence system and quantified using ImageJ. Relative protein abundance was normalized to the corresponding loading control. Detailed antibody information, including catalog numbers and dilution ratios, is provided in Supplementary Table S4.

### Statistical procedures

Computational and statistical analyses were conducted in R (v4.3.1). Associations between continuous variables were determined using Spearman rank correlation. Comparisons involving multiple groups were performed with the Kruskal–Wallis test, whereas pairwise differences were assessed using the Wilcoxon rank-sum test. Multiple-comparison adjustment was implemented according to the Benjamini–Hochberg procedure. For the in vitro functional experiments, comparisons between two groups (e.g., C11orf24 overexpression versus vector control, or C11orf24 knockdown versus sh-NC control) were performed using unpaired two-tailed Student's *t*-tests. Comparisons among three or more groups, including the C11orf24 knockdown and re-expression (rescue) experiments, were analyzed using one-way ANOVA followed by Tukey's post hoc multiple-comparisons test. All functional experiments were independently repeated at least three times (n ≥ 3 independent biological replicates), and quantitative data are presented as mean ± SD. Unless otherwise indicated in the figure legends, significance symbols are defined as follows: ns, not significant (P ≥ 0.05); *P < 0.05; **P < 0.01; ***P < 0.001; ****P < 0.0001. Statistical significance was defined as a two-sided P value below 0.05.

## Results

### Single-cell transcriptomic landscape of bladder cancer reveals distinct epithelial heterogeneity

To dissect the cellular heterogeneity of bladder cancer (BLCA), we performed integrated single-cell transcriptomic analysis of 92,218 cells from 13 treatment-naïve BLCA samples (Fig. 1A, S1A). Unsupervised clustering identified nine major cell lineages, including B cells, endothelial cells, epithelial cells, fibroblasts, mast cells, myeloid cells, plasma cells, proliferating cells (Prolif), and T/NK cells, which were validated by canonical marker gene expression (Fig. 1B–E, S1B). Notably, epithelial cells exhibited substantial inter- and intra-tumoral heterogeneity, with distinct clustering patterns between normal adjacent tissue (NAT) and tumor specimens (Fig. 1B, S1B). Cellular composition analysis revealed marked differences in the abundance of immune and stromal populations across patients with varying clinicopathological characteristics, including lymphovascular invasion status, tumor size, pathological grade, and clinical stage (Fig. 1F). ssGSEA-based pathway enrichment further demonstrated cell-type-specific activation of HALLMARK pathways; for instance, epithelial cells showed enrichment of proliferative and metabolic programs, whereas T/NK cells and myeloid cells were characterized by immune-related signatures (Fig. 1G, S1D–E).

**Fig. 1:**
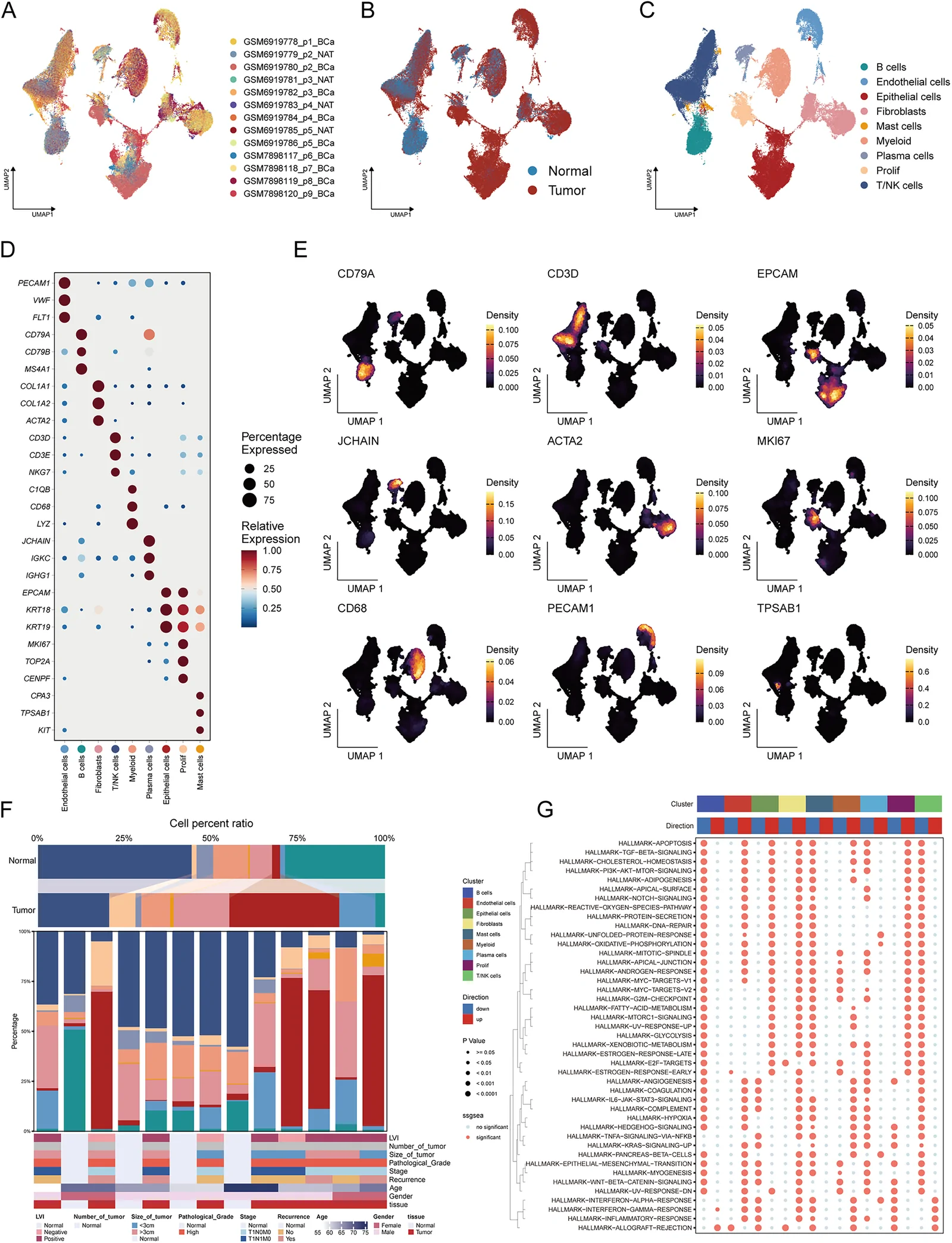
Integrated single-cell transcriptomic landscape of bladder cancer (BLCA). (A) UMAP visualization of 92,218 single cells derived from 13 BLCA samples in GSE222315, colored according to sample origin. (B) UMAP plot illustrating tumor heterogeneity, with cells colored by annotated cell type. (C) Annotation of major cell populations based on canonical marker genes, including B cells, endothelial cells, epithelial cells, fibroblasts, mast cells, myeloid cells, plasma cells, proliferating cells (Prolif), and T/NK cells. (D) Dot plot summarizing the expression of representative marker genes across the nine major cell lineages. Dot size indicates the proportion of expressing cells, whereas color intensity represents average expression levels. (E) Density plots showing the expression distributions of representative marker genes for epithelial cells (EPCAM), B cells (CD79A), plasma cells (JCHAIN), fibroblasts (ACTA2), myeloid cells (CD68), mast cells (TPSAB1), proliferating cells (MKI67), T/NK cells (CD3D), and endothelial cells (PECAM1). (F) Bar plots showing the cellular composition across different tissues (top) and the distribution of cell types across patients and clinicopathological characteristics, including lymphovascular invasion (LVI), tumor number, tumor size, pathological grade, clinical stage, recurrence status, age, sex, and tissue source. (G) ssGSEA-based pathway enrichment analysis highlighting significantly upregulated and downregulated HALLMARK pathways across the nine major cell types.

### Identification of a highly stem-like epithelial subpopulation with distinct copy number and developmental trajectories

Given the pivotal role of epithelial cells in tumor initiation and progression, we focused on delineating their malignant heterogeneity. InferCNV analysis revealed widespread copy number variations (CNVs) across epithelial cells, with clear segregation between normal and tumor-derived populations (Fig. 2A). We first used InferCNV to establish the malignant epithelial compartment based on chromosome-scale copy-number alterations, and then applied CytoTRACE within this compartment to characterize the continuous differentiation and stemness spectrum. Thus, the two analyses served complementary purposes rather than implying that CNV status determines stemness state. To quantify stemness potential, we applied CytoTRACE and identified a continuous spectrum of differentiation states, ranging from highly stem-like to differentiated epithelial cells (Fig. 2B–C). Based on the distribution of CytoTRACE scores, we operationally divided malignant epithelial cells into three stemness states: high-stemness epithelial cells (HStem), differentiation-transition stem-like cells (DTStem), and low-stemness epithelial cells (LStem) (Fig. 2D–F, S2A–C). DTStem cells were retained as an intermediate population in trajectory and comparative analyses, whereas subsequent HStem-associated differential and co-expression analyses primarily contrasted HStem with LStem cells to maximize biological contrast. Pseudotime trajectory analysis reconstructed a developmental hierarchy originating from HStem cells and progressing through DTStem toward terminally differentiated LStem states, with dynamic changes in differentiation-associated gene expression along the trajectory (Fig. 2G–J). Notably, HStem cells were predominantly enriched in advanced-stage tumors (T1N1M0), whereas LStem cells were more prevalent in early-stage lesions (T1N0M0), suggesting stage-dependent stemness evolution (S2B, S2F). The stage-associated differences in HStem and LStem abundance should be interpreted as differences in the distribution of epithelial states across clinical stages rather than evidence that tumor progression reverses or directly follows the inferred pseudotemporal direction. GSEA and scMetabolism analyses revealed that HStem cells were characterized by activation of MYC targets, oxidative phosphorylation, DNA repair, and glycolytic pathways, alongside enhanced metabolic activities in glutathione metabolism and reactive oxygen species signaling (S2D–E).

**Fig. 2:**
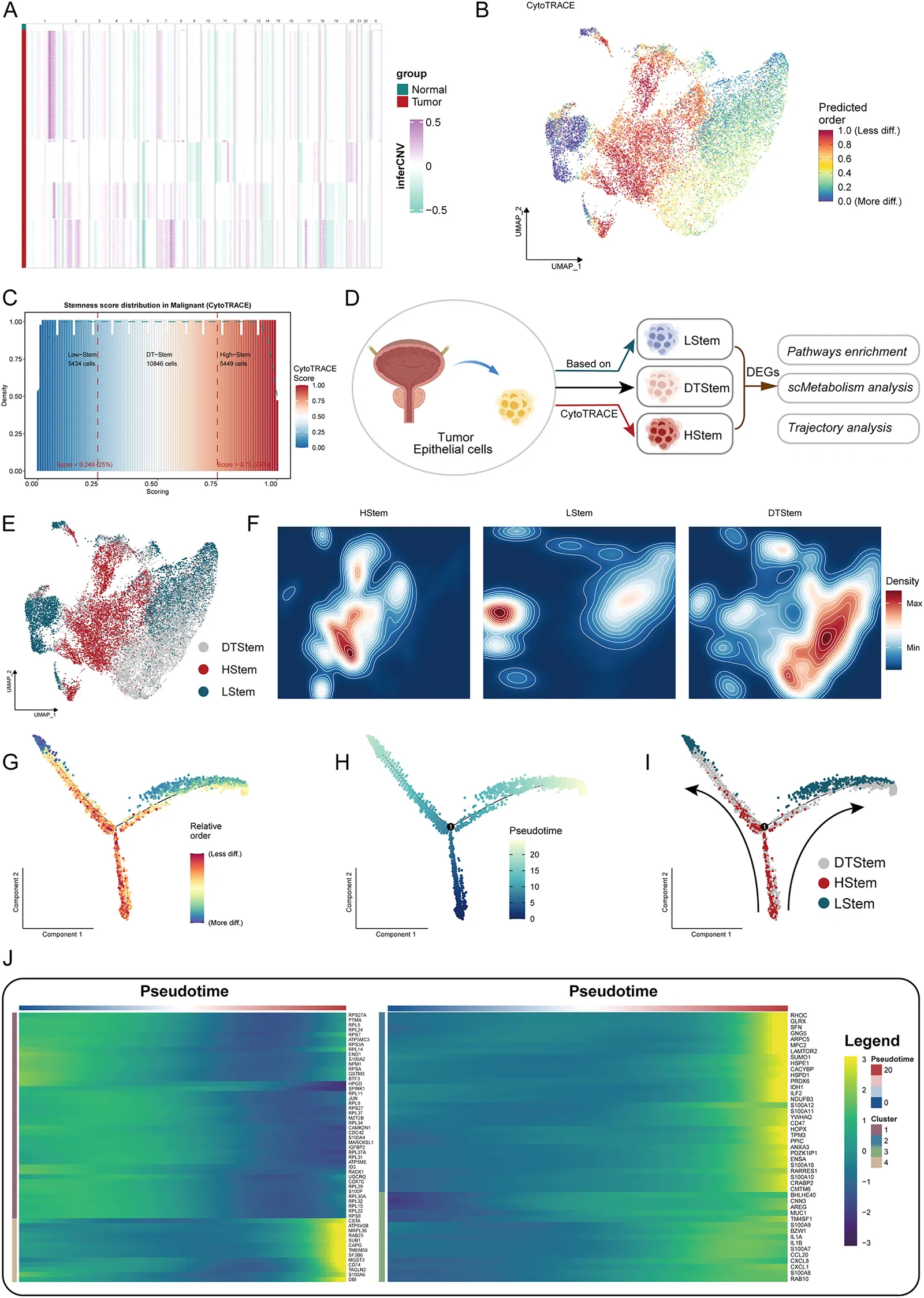
Identification and characterization of stemness-associated epithelial states in BLCA. (A) InferCNV heatmap showing copy number variation (CNV) heterogeneity among epithelial cells, with color intensity representing CNV scores and genomic alterations. (B) Feature plot displaying the distribution of CytoTRACE scores across epithelial cells. (C) Distribution of CytoTRACE scores among epithelial cells. (D) Schematic overview of the functional characterization of the highly stem-like epithelial population (HStem). (E) UMAP visualization of three epithelial subpopulations with distinct stemness states. (F) Density distribution of HStem, DTStem, and LStem subpopulations. (G–I) Pseudotime trajectory analyses depicting the progressive transition from HStem to LStem epithelial cells. (J) Dynamic expression patterns of differentiation-associated genes along the pseudotime trajectory.

### HStem cells engage in specialized intercellular communication with the tumor microenvironment

Cell–cell communication analysis using CellChat revealed that HStem cells exhibited high inferred communication activity within the BLCA microenvironment, exhibiting the highest number of interactions and cumulative interaction strengths among all cellular subsets (Fig. 3A–B, S3A). Comparative analysis between HStem and LStem cells demonstrated that HStem cells showed preferentially higher predicted ligand–receptor interaction probabilities with endothelial cells, fibroblasts, and proliferating cells (Fig. 3C–D). Specifically, HStem cells showed higher predicted outgoing interaction probabilities with fibroblast and endothelial populations, with MIF–CD74/CXCR4 and MIF–CD74/CD44 among the prominent ligand–receptor associations identified in the inferred network (Fig. 3E–F, S3B–F). Differential pathway analysis further revealed that HStem cells were enriched in pro-tumorigenic HALLMARK pathways, including oxidative phosphorylation, adipogenesis, MYC signaling, and reactive oxygen species responses, whereas LStem cells exhibited relatively quiescent phenotypes (Fig. 3H). Spatial transcriptomic analyses demonstrated spatial colocalization of HStem-associated tumor regions with fibroblast- and endothelial-cell populations across multiple samples, providing orthogonal spatial support for the inferred tumor–stroma association (Fig. 3I).

**Fig. 3:**
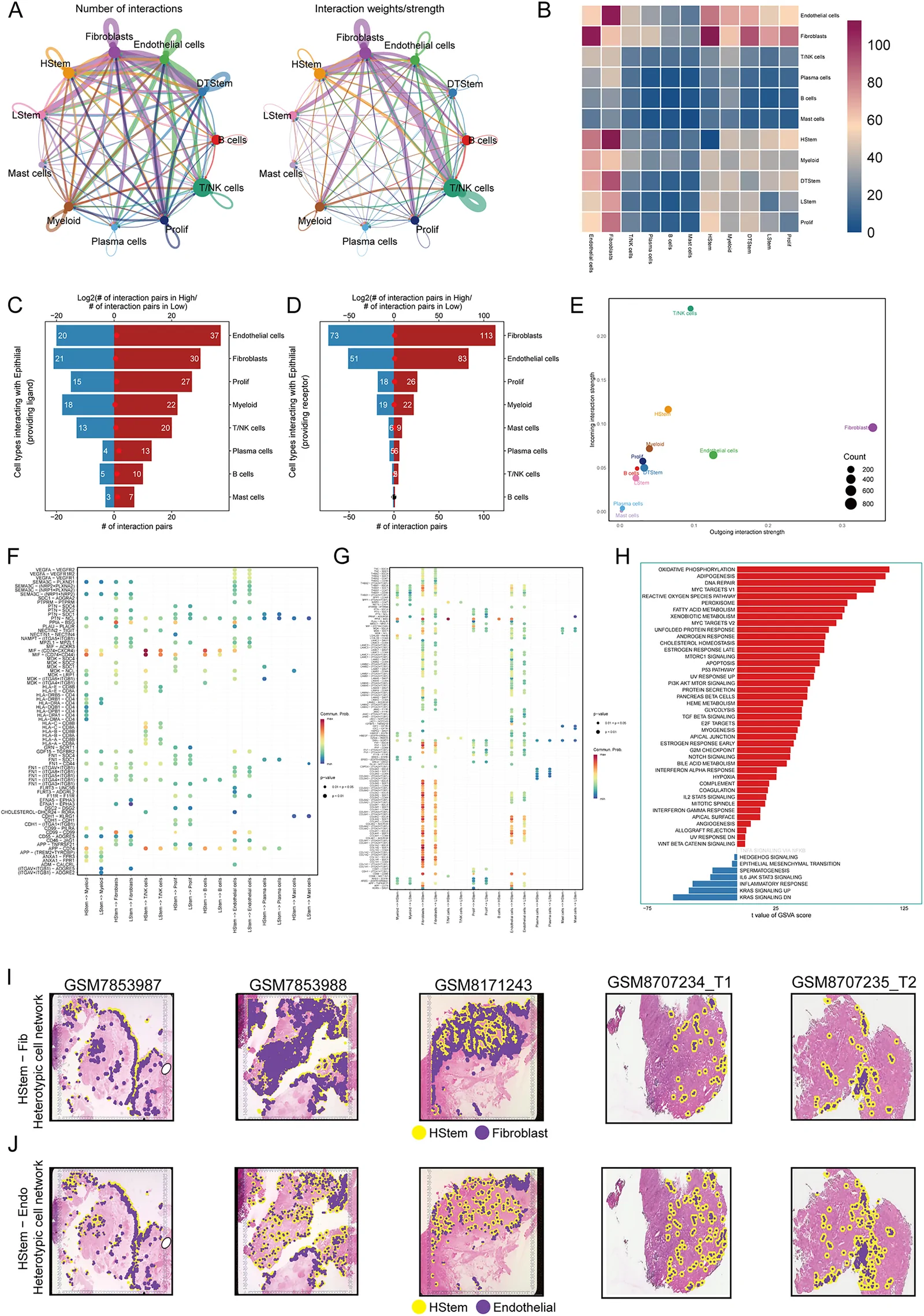
Cell–cell communication networks and functional heterogeneity of stemness-associated epithelial subpopulations. (A) Cell–cell interaction network inferred by CellChat among BLCA cellular subpopulations. (B) Heatmap showing the inferred communication probabilities between different cell populations. (C–D) Comparison of ligand–receptor interactions between HStem and LStem cells, including ligands (C) and receptors (D). (E) Interaction strengths among different cellular populations. (F) Bubble plot showing differential outgoing signaling pathways from HStem and LStem cells. (G) Bubble plot showing differential incoming signaling pathways received by HStem and LStem cells. (H) GSVA analysis comparing HALLMARK pathway activities between HStem and LStem cells. (I) Spatial transcriptomic analyses demonstrating the colocalization of HStem cells with fibroblasts (top) and endothelial cells (bottom) across five representative samples.

### hdWGCNA identifies stemness-associated gene co-expression modules enriched in HStem cells

To uncover the transcriptional programs associated with HStem cell identity, we applied hdWGCNA to malignant epithelial cells and identified six co-expression modules (M1–M6) (Fig. 4A–B). Module activity mapping revealed that M5 and M6 were selectively enriched in HStem cells, whereas M1–M4 showed broader or LStem-biased distributions (Fig. 4C–E). Violin plots confirmed significantly elevated M5 and M6 module scores in HStem compared to DTStem and LStem populations (Fig. 4F). Differential expression analysis between HStem and LStem cells identified 1032 differentially expressed genes, among which 86 upregulated HStem-enriched genes significantly overlapped with M5 and M6 hub genes identified by hdWGCNA (Fig. 4G–H). Disease Ontology enrichment analysis of this stemness-associated signature revealed strong associations with carcinoma-related terms, including urinary bladder cancer, transitional cell carcinoma, and renal cell carcinoma, underscoring the clinical relevance of HStem transcriptional programs (Fig. 4I).

**Fig. 4:**
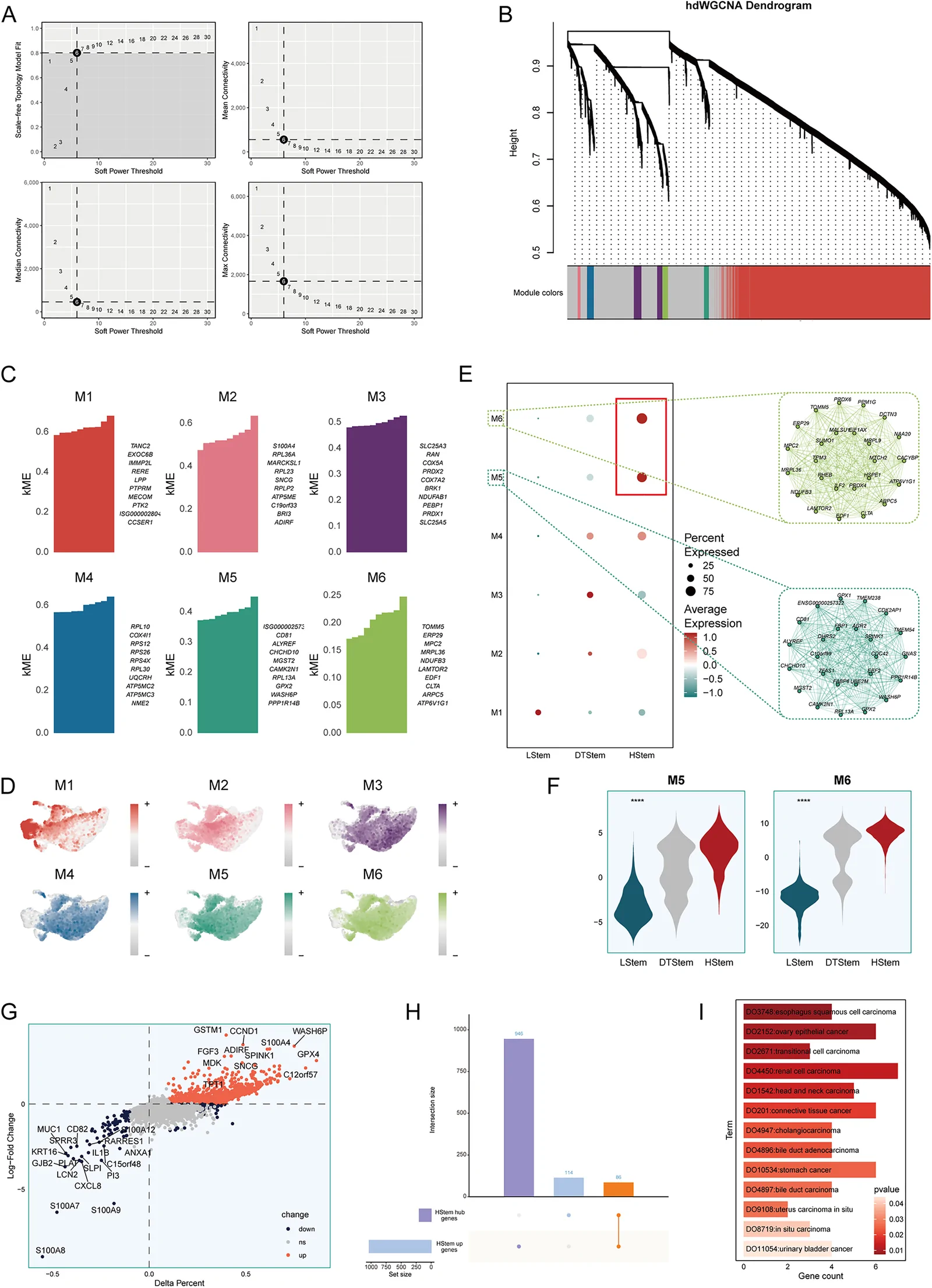
Identification of stemness-associated gene co-expression modules using hdWGCNA. (A) Selection of the optimal soft-thresholding power based on scale-free topology fit and connectivity analyses, identifying power = 6 as the optimal parameter. (B) Hierarchical clustering dendrogram of gene co-expression modules identified by hdWGCNA, revealing six modules (M1–M6). (C) Top 10 hub genes for each module (M1–M6). (D) UMAP visualization of module activities across epithelial cell subpopulations. (E) Dot plot illustrating module gene expression patterns among stemness-associated epithelial subpopulations, highlighting enrichment of M5 and M6 in HStem cells. (F) Violin plots showing elevated M5 and M6 module activities in HStem cells. (G) Differential expression analysis between HStem and LStem cells. (H) Overlap between HStem hub genes and upregulated genes, yielding 86 stemness-associated signature genes. (I) Disease Ontology (DO) enrichment analysis of the identified signature genes.

### Development and validation of an HStem-associated image-based prognostic model

Leveraging the HStem signature, we developed an integrative prognostic framework combining histopathological image features with machine learning. The pipeline involved whole-slide image (WSI) patch extraction, CellProfiler-based pathological feature quantification, and ResNet-50 deep learning feature extraction, followed by correlation-based feature selection (Fig. 5A). A comprehensive benchmarking framework evaluating 101 algorithm combinations identified StepCox(forward) + Ridge as the optimal model, which was subsequently validated in independent BLCA cohorts (Fig. 5B–D). Univariate Cox regression identified 17 robust prognostic image features, including Total_Granularity_8_OrigGray and multiple ResNet-derived deep features (Fig. 5C). The model-development cohort included 298 patients, including 139 deaths/events, whereas the validation cohort included 76 patients, including 31 deaths/events. The optimized risk score demonstrated strong prognostic discrimination, with high-risk patients exhibiting significantly inferior overall survival in both training and validation cohorts (Fig. 5E–F). Restricted cubic spline analysis revealed a non-linear, monotonic relationship between increasing risk scores and higher hazard ratios, with the curve exhibiting a steep upward trajectory at higher risk score values (Fig. 5G). Multivariate Cox regression established the risk score as an independent prognostic factor (HR = 1.68, 95% CI: 1.08–2.60, P = 0.022) after adjusting for age, N stage, M stage, and clinical stage (Fig. 5H–I). Calibration curves and decision curve analyses further supported the model's predictive accuracy and clinical utility (S4A–C).

**Fig. 5:**
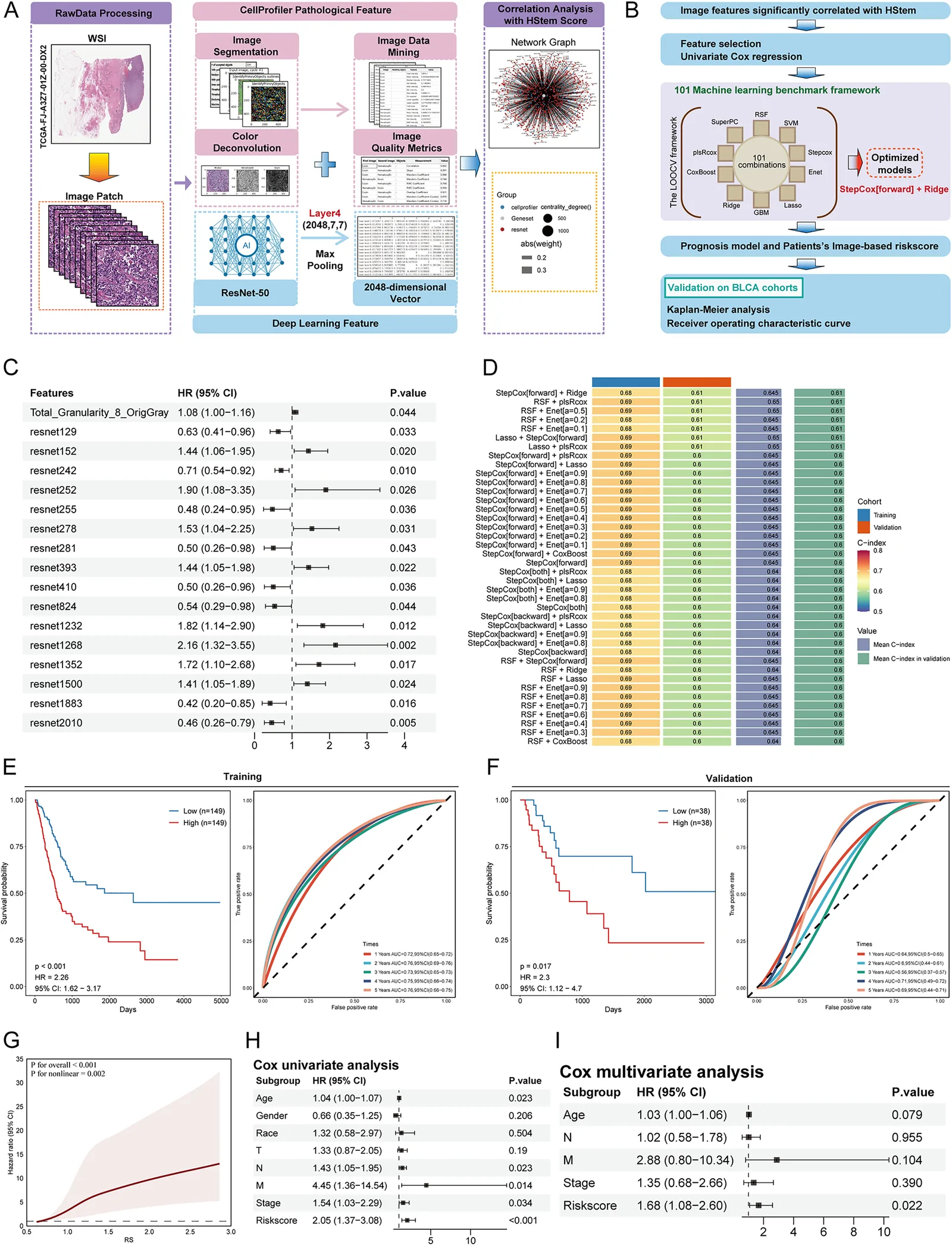
Construction and validation of an HStem-associated image-based prognostic model. (A) Workflow of histopathological image feature extraction. (B) Machine-learning benchmarking framework used for model development. (C) Identification of robust prognostic image features using univariate Cox regression analysis. (D) Screening of the optimal predictive model from 101 machine-learning algorithms. (E–F) Kaplan–Meier survival analyses and time-dependent ROC curves evaluating the prognostic performance of the model in the training and validation cohorts. (G) Restricted cubic spline analysis illustrating the relationship between risk scores and hazard ratios in the Cox proportional hazards model. (H–I) Forest plots of univariate and multivariate Cox regression analyses integrating clinical variables and risk scores.

### HStem-derived risk score correlates with immune exclusion and predicted immunotherapy resistance

We next investigated the relationship between HStem risk scores and the tumor immune microenvironment (TIME). High-risk tumors displayed significantly reduced infiltration of cytotoxic immune populations, including CD8+ T cells, NK cells, and activated dendritic cells, together with increased abundance of immunoregulatory populations such as regulatory T cells (Tregs) and myeloid-derived suppressor cells (MDSCs) (Fig. 6A). Features associated with immune exclusion, including fibroblast-associated programs and epithelial–mesenchymal transition (EMT) signatures, were enriched in high-risk tumors, consistent with a microenvironment in which immune-cell access may be spatially constrained by stromal remodeling (Fig. 6B). Separately, immunosuppressive features were characterized by enrichment of Treg-associated markers, immune checkpoint molecules, and T-cell dysfunction signatures (Fig. 6C–D). Thus, the high-risk phenotype exhibited evidence of both immune exclusion and immunosuppression, which were considered related but biologically distinct properties of the tumor immune microenvironment. TIDE analysis indicated enhanced predicted immune evasion potential in high-risk patients, suggesting compromised responses to immune checkpoint blockade (ICB) therapy (Fig. 6E). Histopathological examination revealed that high-risk tumors were characterized by absent or sparse lymphocytic infiltration, whereas low-risk tumors exhibited prominent lymphocyte-rich stromal responses (Fig. 6F). Integrated analysis of immune checkpoint ligand–receptor interactions and genomic alterations across risk groups revealed differential expression of co-stimulatory and co-inhibitory molecules, with high-risk tumors showing downregulation of antigen presentation machinery and upregulation of immune evasion pathways (Fig. 6G).

**Fig. 6:**
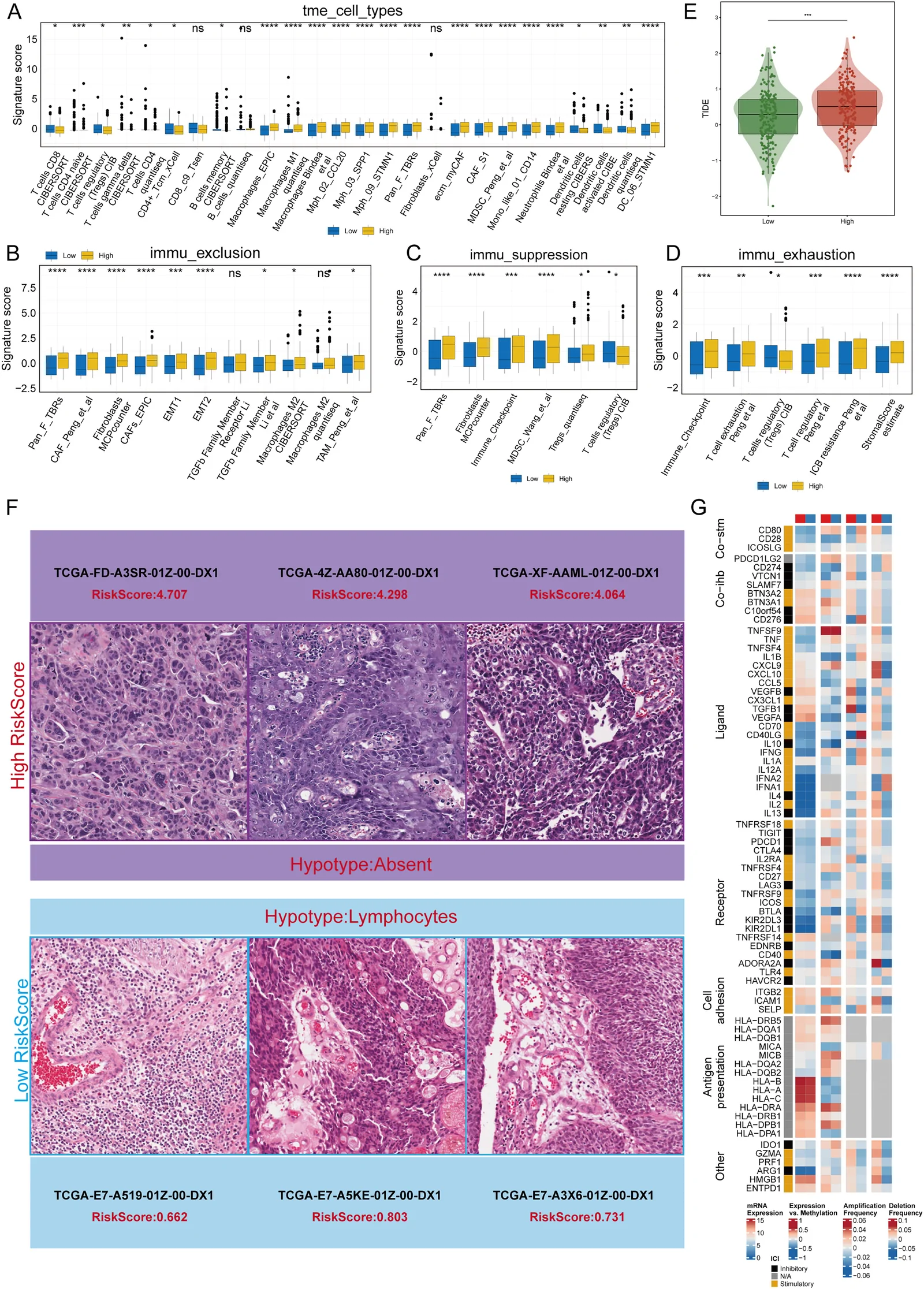
Association of the risk score with the tumor immune microenvironment and therapeutic response. (A) Comparison of tumor-infiltrating immune cell (TIIC) abundance between risk groups. (B) Immune exclusion analysis highlighting fibroblast-associated pathways and epithelial–mesenchymal transition (EMT) signatures. (C) Enrichment of immune exhaustion features in high-risk patients, including regulatory T cells (Tregs) and suppressed immune checkpoint blockade (ICB) responses. (D) Immunosuppressive characteristics enriched in the high-risk group, including Tregs and immune checkpoint expression. (E) TIDE analysis indicating enhanced immune evasion in high-risk patients. (F) Histopathological validation demonstrating increased immune infiltration in low-risk tumors. (G) Integrated landscape of immune checkpoint ligand–receptor interactions and genomic alterations across risk groups.

To identify potential therapeutic vulnerabilities associated with the HStem phenotype, we performed computational drug sensitivity prediction using the Genomics of Drug Sensitivity in Cancer (GDSC) database, which quantifies drug response through half-maximal inhibitory concentration (IC50) values derived from dose-response profiling across cancer cell lines. High-risk patients were predicted to exhibit significantly enhanced sensitivity to dasatinib, AZD8055, and luminespib, as evidenced by lower estimated IC50 values compared to low-risk patients (S4D). Complementary analysis using the Cancer Therapeutics Response Portal (CTRP) and PRISM (Profiling Relative Inhibition Simultaneously in Mixtures) databases, which profile compound sensitivity through area-under-the-curve (AUC) metrics, revealed additional agents with predicted preferential activity in high-risk tumors (S4E). These convergent profiles from independent pharmacogenomic resources suggest that HStem-high tumors may harbor distinct predicted drug sensitivities. Because these analyses provide computationally predicted rather than experimentally demonstrated drug responses, these findings should be regarded as hypothesis-generating therapeutic vulnerabilities that require experimental validation before any translation into precision therapeutic strategies targeting the stem-like epithelial compartment.

### C11orf24 emerges as an HStem-associated hub gene with oncogenic functions

Among the six HStem hub genes (C11orf24, CANX, VEGFB, ARF6, ALYREF, and EIF3A), C11orf24 showed the strongest positive association with the HStem-associated risk score and exhibited prominent connectivity with prognostic histopathological features (Fig. 7A–B). Correlation analysis identified associations between C11orf24 expression and several pathological image features, including resnet278 and resnet242 (Fig. 7C–D). The P values presented in Fig. 7C are nominal two-sided Spearman correlation P values without multiple-testing adjustment and are therefore interpreted as exploratory. Expression analysis across disease stages revealed significantly elevated C11orf24, CANX, VEGFB, ARF6, ALYREF, and EIF3A in N1 compared to N0 tumors (Fig. 7E). ROC curve analysis in the TCGA-BLCA cohort confirmed the diagnostic potential of these hub genes for distinguishing tumor from normal tissues (Fig. 7F). In the TCGA-BLCA cohort, the majority of these six hub genes were significantly upregulated in tumor versus normal tissues, a finding validated in paired sample analyses (Fig. 7G–I, S5A–B). Kaplan–Meier survival analyses demonstrated that high C11orf24 expression was consistently associated with poor prognosis in both the TCGA-BLCA and GSE31684 cohorts (Fig. 7J). Notably, C11orf24 expression positively correlated with multiple immune infiltration features and oncogenic CancerSEA functional states, including EMT, invasion, metastasis, proliferation, and stemness (S5C–E).

**Fig. 7:**
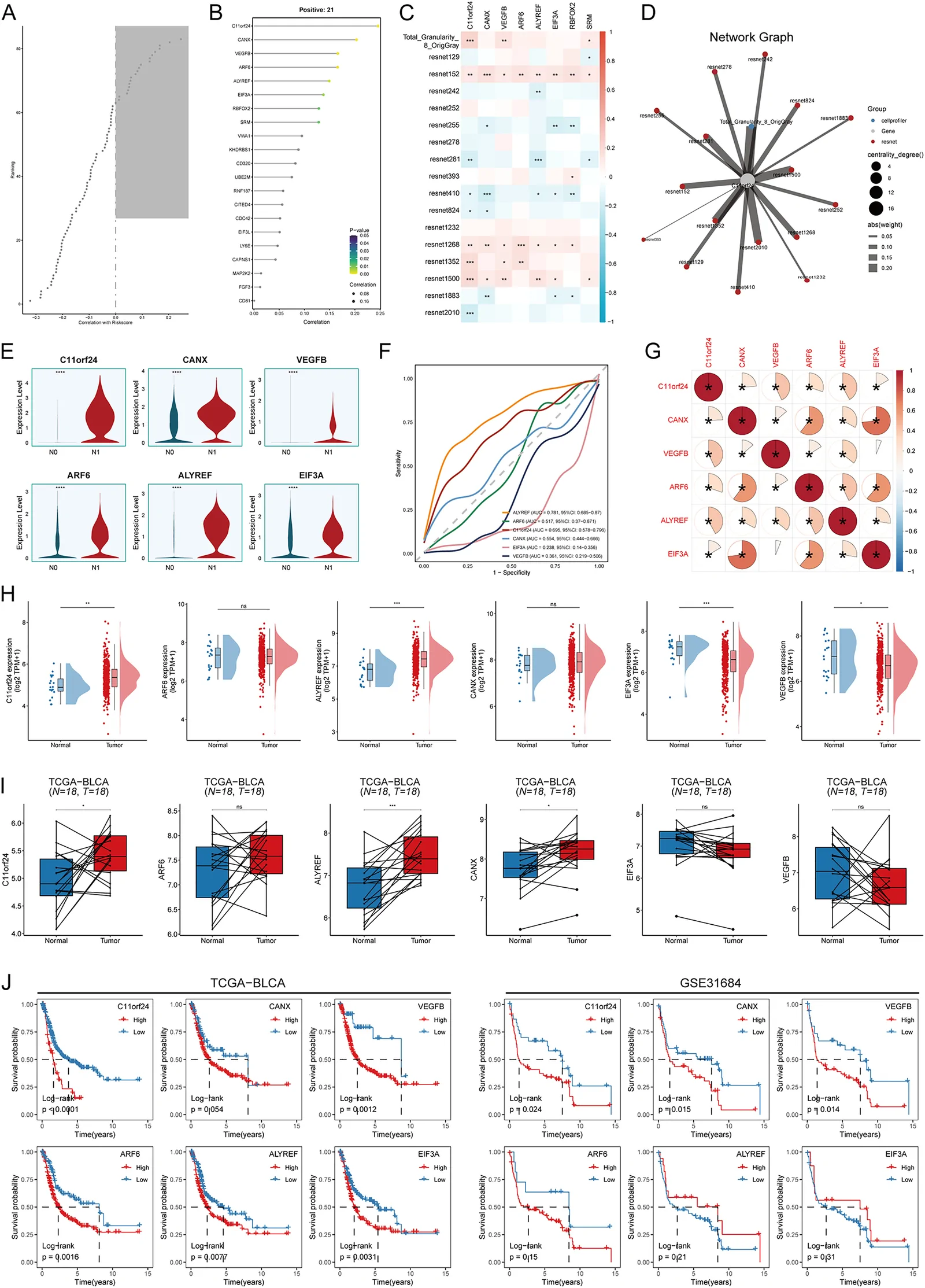
Identification, validation, and clinical relevance of HStem-associated hub genes. (A) Correlation analysis between HStem signature genes and the HStem-associated risk score. (B) Lollipop plot showing HStem signature genes positively correlated with the risk score. (C) Correlation analysis between the top eight most significant genes and key pathological image features. P values are nominal two-sided Spearman correlation P values without multiple-testing adjustment and should be interpreted as exploratory. (D) Network visualization illustrating the association between the most strongly correlated gene (C11orf24) and key image features. (E) Violin plots showing the expression levels of C11orf24, CANX, VEGFB, ARF6, ALYREF, and EIF3A in epithelial cells from N0 and N1 disease stages. (F) ROC curves evaluating the diagnostic performance of the six hub genes. (G) Correlation heatmap of the six hub genes in the TCGA-BLCA cohort. (H) Integrated violin, box, and scatter plots comparing gene expression between normal and tumor tissues. (I) Paired boxplots showing differential gene expression between matched normal and tumor samples. (J) Kaplan–Meier survival analyses of six hub genes in the TCGA-BLCA and GSE31684 cohorts.

### C11orf24 defines a malignant epithelial subpopulation with enhanced stemness and aggressive signaling

To characterize C11orf24+ epithelial cells at single-cell resolution, we stratified malignant epithelial cells into C11orf24-positive (C11orf24+ Epi) and C11orf24-negative (C11orf24− Epi) subsets (Fig. 8A–B). GSEA revealed that C11orf24+ Epi cells were significantly enriched in MYC targets, oxidative phosphorylation, DNA repair, chemical carcinogenesis, glycolysis, and glutathione metabolism (Fig. 8C, S5D). CytoTRACE analysis confirmed higher stemness scores in C11orf24+ Epi cells, with a significant positive correlation between C11orf24 expression and stemness potential (Fig. 8D–E). Slingshot trajectory inference positioned C11orf24+ Epi cells at the root of the developmental hierarchy, with progressive loss of C11orf24 expression along pseudotime (Fig. 8F–G). PySCENIC analysis identified distinct transcription factor regulatory networks, with C11orf24+ Epi cells showing activation of FOXA1, JUND, and FOS, whereas C11orf24− Epi cells were characterized by SOX4 and SPI1 activity (Fig. 8H–I). CellChat analysis revealed that C11orf24+ Epi cells preferentially communicated with fibroblasts, endothelial cells, and myeloid cells (Fig. 8J, S3J). Spatial transcriptomics confirmed widespread C11orf24 expression across tumor regions (Fig. 8K). MistyR spatial analysis demonstrated that C11orf24+ Epi cells colocalized with fibroblasts and endothelial cells within the intra-tumoral niche, whereas C11orf24− Epi cells were spatially associated with B cells and T/NK cells (Fig. 8L–M). CancerSEA pathway analysis further revealed that C11orf24+Epi cells colocalized with stemness, proliferation, metastasis, and angiogenesis pathways compared to C11orf24−Epi cells (Fig. 8N).

**Fig. 8:**
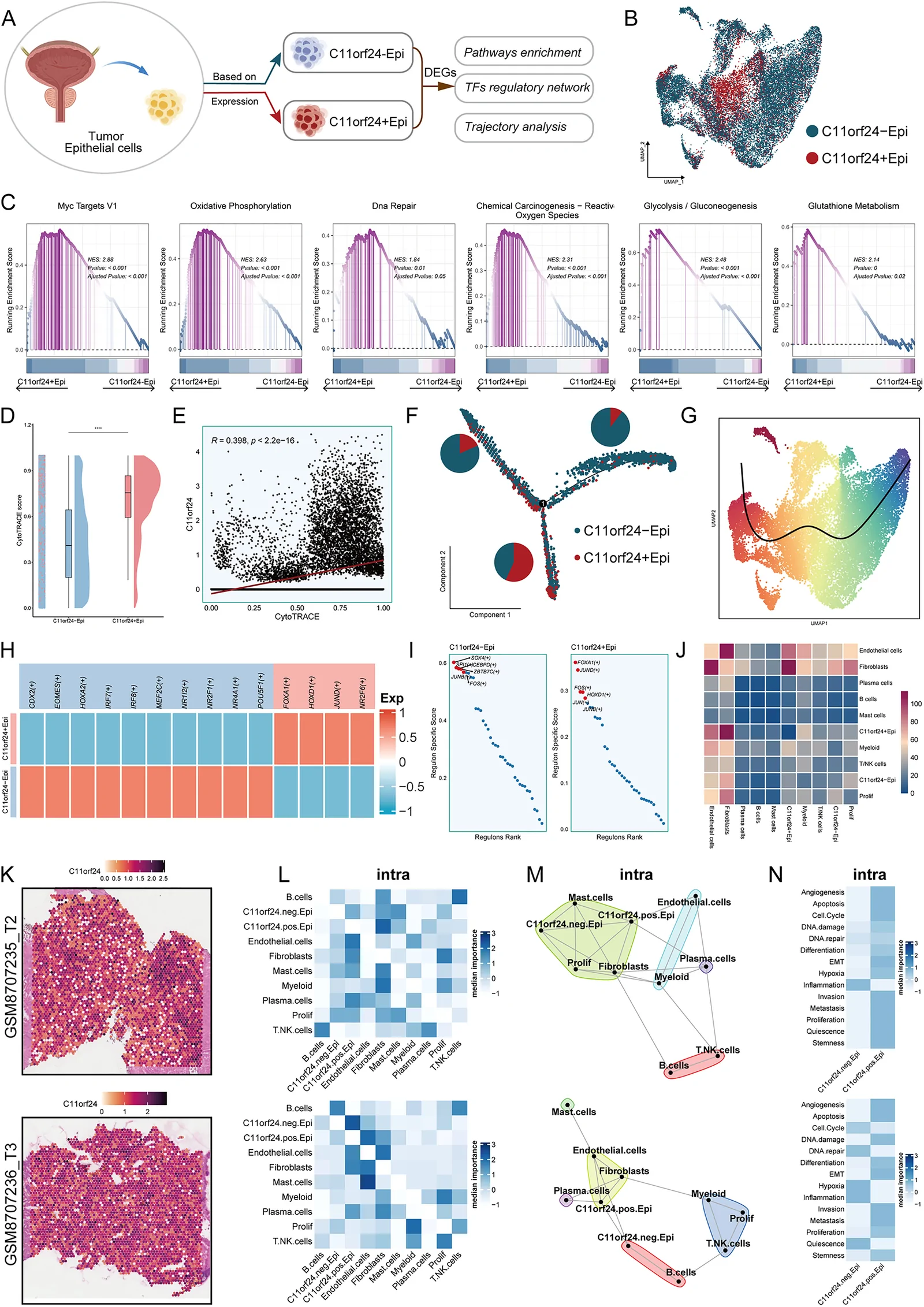
Functional and regulatory characteristics of C11orf24⁺ epithelial cells. (A) Schematic overview of C11orf24-positive epithelial cells (C11orf24⁺ Epi). (B) UMAP visualization of C11orf24⁺ and C11orf24⁻ epithelial cells. (C) GSEA comparing pathway enrichment between C11orf24⁺ and C11orf24⁻ epithelial cells. (D) Raincloud plot comparing CytoTRACE scores between C11orf24⁺ and C11orf24⁻ epithelial cells. (E) Correlation between C11orf24 expression and CytoTRACE scores. (F) Dynamic changes in cell-state proportions along the developmental trajectory. (G) Slingshot-inferred developmental trajectory colored by pseudotime progression. (H) PySCENIC heatmap showing differential transcription factor activities. (I) Transcription factor regulatory network analysis. (J) CellChat-based communication heatmap. (K) Spatial expression patterns of C11orf24 in representative samples. (L) Within-region cellular colocalization heatmap generated by MistyR. (M) Cell dependency network inferred by MistyR. (N) Spatial proximity heatmap between C11orf24-defined epithelial subpopulations and CancerSEA functional states.

### Experimental validation supports the oncogenic role of C11orf24 in bladder cancer cells

To functionally validate C11orf24, we performed gain- and loss-of-function studies in EJ and T24 BLCA cell lines. RT-qPCR confirmed efficient C11orf24 overexpression and knockdown (sh#1 and sh#2) in both cell lines (Fig. 9A–B). Colony formation assays demonstrated that C11orf24 overexpression significantly enhanced clonogenic capacity, whereas C11orf24 knockdown markedly suppressed colony formation (Fig. 9C–F). Wound-healing assays revealed that C11orf24 overexpression accelerated migratory capacity, with wound closure rates increasing from ∼30% to ∼55% in EJ cells and from ∼45% to ∼75% in T24 cells; conversely, C11orf24 knockdown impaired migration in both cell lines (Fig. 9G–J). Transwell invasion assays further confirmed that C11orf24 overexpression significantly increased the number of migrated and invaded cells, whereas C11orf24 silencing suppressed invasive potential (Fig. 9K–O). Collectively, these findings indicate that C11orf24 functionally promotes the proliferation, migration, and invasion of BLCA cells in vitro. To further examine whether C11orf24 is associated with the stemness phenotype identified by our single-cell analyses, we evaluated canonical stemness-associated markers following C11orf24 perturbation. In EJ cells, C11orf24 overexpression increased the mRNA levels of SOX2, ALDH1A1, and CD44, accompanied by increased protein expression of these markers (Fig. 10A,C). Conversely, C11orf24 knockdown in T24 cells reduced SOX2, ALDH1A1, and CD44 at both the transcript and protein levels (Fig. 10B,D). Importantly, re-expression of C11orf24 in C11orf24-silenced T24 cells partially restored the expression of these stemness-associated markers (Fig. 10B,D). Together, these findings provide experimental support for an association between C11orf24 expression and the stemness-associated phenotype of BLCA cells.

**Fig. 9:**
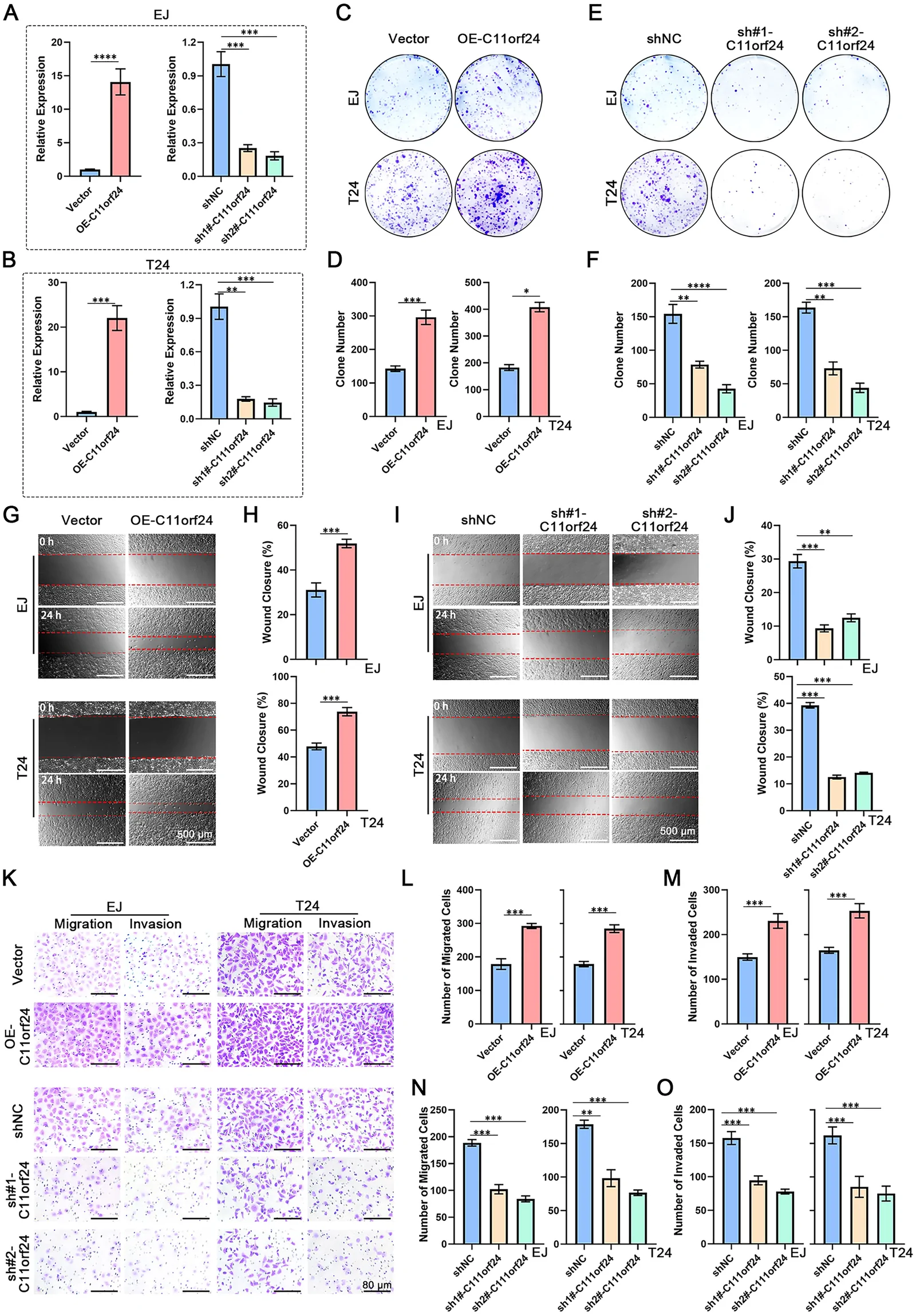
Experimental validation of the oncogenic role of C11orf24 in bladder cancer cells. (A) RT-qPCR analysis of C11orf24 mRNA expression following C11orf24 overexpression or knockdown in EJ cells. (B) RT-qPCR analysis of C11orf24 mRNA expression following C11orf24 overexpression or knockdown in T24 cells. (C–D) Representative images of colony formation assays performed following C11orf24 overexpression in EJ and T24 cells (C), with quantitative analysis shown in (D). Statistical significance was determined using an unpaired Student’s *t*-test. Data are presented as mean ± SD. ns, not significant; *P < 0.05; **P < 0.01; ***P < 0.001; ****P < 0.0001. (E–F) Representative images of colony formation assays performed following C11orf24 knockdown in EJ and T24 cells (E), with quantitative analysis shown in (F). Statistical significance was determined using an unpaired Student’s *t*-test. Data are presented as mean ± SD. ns, not significant; *P < 0.05; **P < 0.01; ***P < 0.001; ****P < 0.0001. (G–H) Wound-healing assays evaluating the migratory capacity of EJ and T24 cells following C11orf24 overexpression (G), with quantitative analysis shown in (H). Statistical significance was determined using an unpaired Student’s *t*-test. Data are presented as mean ± SD. ns, not significant; *P < 0.05; **P < 0.01; ***P < 0.001; ****P < 0.0001. (I–J) Wound-healing assays evaluating the migratory capacity of EJ and T24 cells following C11orf24 knockdown (I), with quantitative analysis shown in (J). Statistical significance was determined using an unpaired Student’s *t*-test. Data are presented as mean ± SD. ns, not significant; *P < 0.05; **P < 0.01; ***P < 0.001; ****P < 0.0001. (K–O) Transwell invasion assays performed following C11orf24 overexpression or knockdown in EJ and T24 cells (K), with quantitative analyses shown in (L–O). Statistical significance was determined using an unpaired Student’s *t*-test. Data are presented as mean ± SD. ns, not significant; *P < 0.05; **P < 0.01; ***P < 0.001; ****P < 0.0001.

**Fig. 10:**
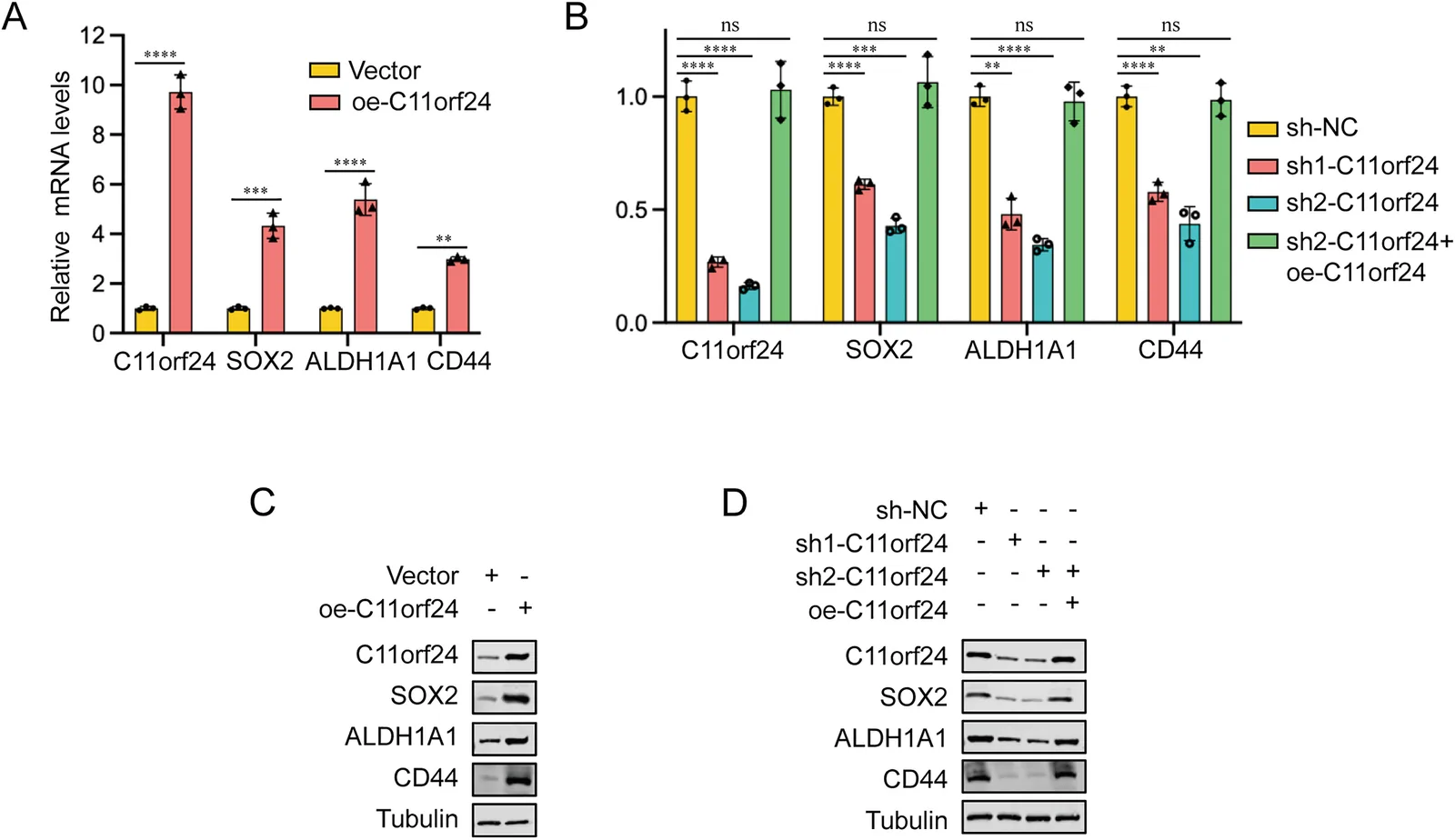
C11orf24 positively regulates cancer stemness marker expression in bladder cancer cells. (A) RT-qPCR analysis showing relative mRNA levels of C11orf24, SOX2, ALDH1A1, and CD44 in EJ cells overexpressing C11orf24 (oe-C11orf24) compared to vector control. (B) RT-qPCR analysis of C11orf24, SOX2, ALDH1A1, and CD44 mRNA levels in T24 cells with C11orf24 knockdown (sh1-C11orf24 and sh2-C11orf24) and rescue experiments (sh2-C11orf24 + oe-C11orf24) compared to sh-NC control. (C) Western blot analysis of C11orf24, SOX2, ALDH1A1, and CD44 protein levels in EJ cells overexpressing C11orf24. (D) Western blot analysis of C11orf24, SOX2, ALDH1A1, and CD44 protein levels in T24 cells with C11orf24 knockdown and rescue. Tubulin served as a loading control. Statistical significance was determined using unpaired two-tailed Student's *t*-tests for two-group comparisons and one-way ANOVA followed by Tukey's post hoc test for comparisons among three or more groups. Data are presented as mean ± SD from at least three independent biological replicates. ns, not significant; *P < 0.05; **P < 0.01; ***P < 0.001; ****P < 0.0001.

### C11orf24-positive epithelial cells exhibit predicted therapeutic vulnerabilities

To identify compounds with predicted selective activity against the C11orf24+ stem-like epithelial compartment, we performed single-cell drug sensitivity screening using the BeyondCell framework. Differential sensitivity pattern analysis across the transcriptomic landscape identified CEPHAELINE (sig-17,814) and SPIRONOLACTONE (sig-20,845) as top candidate compounds with predicted preferential sensitivity in C11orf24+Epi cells (S6A–B). BeyondCell scoring confirmed that C11orf24+Epi cells exhibited significantly higher drug sensitivity scores compared to C11orf24−Epi cells for both compounds (CEPHAELINE: mean BCS = 24.43 vs. −8.73, P < 0.001; SPIRONOLACTONE: mean BCS = 7.72 vs. −11.28, P < 0.001), indicating higher predicted sensitivity within the stem-like compartment (S6C). UMAP feature plots localized drug response signatures to HStem-enriched regions, and violin plots confirmed preferential predicted sensitivity of HStem relative to LStem populations (S6D–E). These pharmacogenomic predictions are hypothesis-generating: they nominate candidate therapeutic vulnerabilities of the C11orf24-positive stem-like epithelial compartment that must be validated experimentally in preclinical models before any translational application.

## Discussion

Cancer stem-like cells are increasingly recognized as key drivers of tumor initiation, progression, therapeutic resistance, and disease recurrence[20,21]. However, the cellular hierarchy underlying stemness programs in bladder cancer (BLCA) remains incompletely understood. In this study, by integrating single-cell transcriptomics, spatial transcriptomics, computational pathology, and experimental validation, we identified a previously uncharacterized highly stem-like epithelial population (HStem) that occupies the apex of the malignant epithelial differentiation hierarchy. We further showed that HStem cells exhibit extensive predicted interactions with the tumor microenvironment (TME), are associated with immune exclusion and immunotherapy resistance, and give rise to a clinically applicable image-based prognostic model. In addition, we identified C11orf24 as a candidate regulator associated with the HStem phenotype and experimentally validated its oncogenic functions in BLCA cells. Together, these findings provide a conceptual framework linking epithelial stemness, microenvironmental remodeling, and clinical outcomes in BLCA.

Intratumoral heterogeneity represents a major obstacle to effective cancer treatment[22,23]. Although previous single-cell studies have revealed substantial cellular diversity in BLCA, the developmental relationships among malignant epithelial subpopulations and their contributions to disease progression remain poorly defined. By combining inferCNV, CytoTRACE, and pseudotime analyses, we identified a continuous differentiation trajectory extending from HStem cells through intermediate states toward terminally differentiated epithelial populations. Notably, HStem cells preferentially accumulated in advanced-stage tumors and displayed activation of MYC signaling, oxidative phosphorylation, DNA repair, glycolysis, and glutathione metabolism. These findings are consistent with emerging evidence suggesting that cancer stem-like cells exhibit enhanced metabolic plasticity and stress adaptation capabilities, enabling them to survive hostile microenvironmental conditions and sustain tumor evolution[24,25]. Our results therefore support a hierarchical model in which stem-like epithelial cells serve as a reservoir for tumor progression and phenotypic diversification in BLCA.

Beyond their intrinsic transcriptional programs, HStem cells appeared to be closely associated with the surrounding microenvironment. CellChat and spatial transcriptomic analyses consistently demonstrated that HStem cells were inferred to function as major communication hubs within the tumor ecosystem, exhibiting particularly strong interactions with fibroblasts and endothelial cells[26,27]. Among the identified signaling pathways, MIF–CD74/CXCR4 and MIF–CD74/CD44 signaling emerged as prominent predicted communication axes. MIF signaling has previously been implicated in tumor-promoting inflammation, angiogenesis, stemness maintenance, and immune suppression across multiple cancer types[28]. Our findings suggest that these MIF-related interactions may contribute to the association between HStem states and stromal niches, although their functional dependence and causal roles remain to be established. The spatial colocalization of HStem cells with fibroblasts and endothelial cells further supports the existence of specialized stemness-supportive niches within BLCA, analogous to cancer stem cell niches described in other solid tumors[29,30].

A notable aspect of our study is the integration of single-cell-derived biological insights with computational pathology. While most stemness studies rely on transcriptomic biomarkers, their clinical implementation is often limited by the requirement for molecular profiling. To address this challenge, we developed an image-based prognostic model derived from HStem-associated transcriptional programs and histopathological features. This model demonstrated robust prognostic performance across independent cohorts and remained an independent predictor after adjustment for established clinicopathological variables[31]. These findings highlight the potential of computational pathology to bridge molecular discoveries and routine clinical practice, enabling non-invasive assessment of stemness-associated risk directly from standard hematoxylin and eosin-stained slides[32]. Such an approach may facilitate broader clinical deployment of stemness-informed precision oncology strategies.

The translational implications of targeting stem-like tumor populations should nevertheless be considered cautiously. Cancer stem-like states are highly plastic and may dynamically transition in response to therapeutic pressure and microenvironmental cues. Consequently, broad elimination of stemness-associated populations may not be sufficient and could potentially select for adaptive or resistant cellular states. The pharmacogenomic findings in the present study should therefore be considered hypothesis-generating rather than evidence of established therapeutic efficacy. They may provide a starting point for identifying vulnerabilities associated with the C11orf24-positive/HStem phenotype that can be tested in appropriate preclinical models. Similarly, although C11orf24-positive epithelial cells showed enrichment of MYC-related and metabolic programs, these observations demonstrate an association rather than a direct regulatory relationship between C11orf24 and MYC. Direct molecular perturbation and rescue experiments will be required to determine whether MYC or specific metabolic pathways mediate the effects of C11orf24. From a clinical perspective, the image-based model has the potential to be incorporated into digital pathology workflows using routinely available H&E-stained slides. However, its clinical implementation would require prospective, multicenter validation, standardized slide acquisition and preprocessing, and assessment of performance across scanners, institutions, and patient populations.

An additional important observation is the close association between HStem-related risk and an immune-excluded and immunosuppressive tumor microenvironment. High-risk tumors exhibited reduced infiltration of cytotoxic immune populations, including CD8+ T cells and NK cells, together with increased abundance of Tregs, MDSCs, and fibroblast-associated programs. Furthermore, TIDE analysis indicated enhanced immune evasion and reduced likelihood of response to immune checkpoint blockade (ICB). These findings suggest that stemness may represent a central biological process linking tumor-intrinsic plasticity and immune escape. Recent studies have proposed that cancer stem-like cells can evade immune surveillance through impaired antigen presentation, induction of T-cell dysfunction, and recruitment of immunosuppressive stromal populations[33,34]. Our data extend these observations to BLCA and suggest that HStem-high tumors may constitute an immunologically “cold” phenotype characterized by both restricted immune-cell infiltration and immunosuppressive immune programs, together with a predicted lower likelihood of response to immunotherapy. Importantly, the predicted drug sensitivity profiles suggest that stemness-associated vulnerabilities may represent candidate therapeutic opportunities that warrant experimental validation in these patients[35]^.^

Among the HStem-associated genes, C11orf24 emerged as a particularly compelling candidate. Importantly, perturbation of C11orf24 altered the expression of SOX2, ALDH1A1, and CD44, providing experimental support for its association with stemness-related molecular features. Although C11orf24 has received limited attention in cancer biology, our analyses consistently identified it as a central hub linking stemness programs, pathological image features, disease progression, and clinical outcome[36]. At single-cell resolution, C11orf24-positive epithelial cells occupied the earliest developmental states, exhibited elevated CytoTRACE scores, activated MYC-related and metabolic pathways, and preferentially interacted with fibroblasts, endothelial cells, and myeloid cells. Functional experiments further demonstrated that C11orf24 promotes proliferation, migration, invasion, and clonogenicity in BLCA cells. Collectively, these findings support C11orf24 as a BLCA-associated candidate linked to malignant epithelial stemness and suggest its potential utility as a prognostic biomarker and candidate therapeutic target. However, its broader role across cancer types remains insufficiently characterized and requires further mechanistic investigation. Moreover, the identification of selective therapeutic vulnerabilities in C11orf24-positive epithelial cells raises the possibility of targeting stem-like tumor compartments that are often responsible for recurrence and treatment failure. However, these findings do not establish bona fide self-renewal or lineage-plasticity functions, which would require sphere-formation assays, functional ALDH activity assays, lineage-tracing approaches, and/or in vivo validation.

Several limitations should be acknowledged. First, although our analyses integrated multiple orthogonal datasets and included experimental validation, the majority of mechanistic inferences regarding HStem-mediated cell–cell communication remain computational and require further validation in vivo. Second, the primary single-cell analysis was based on GSE222315, comprising 92,218 cells from 13 treatment-naïve BLCA samples. Although independent spatial transcriptomic datasets and bulk cohorts provided orthogonal validation, these datasets do not substitute for an independent single-cell replication cohort. Therefore, the reproducibility of the HStem/DTStem/LStem classification across independent scRNA-seq cohorts remains to be established. Third, the functional experiments were performed primarily in EJ and T24 BLCA cell lines. Although the concordant gain- and loss-of-function phenotypes across these two independent cellular backgrounds provide internal experimental consistency, additional BLCA models representing different molecular subtypes and differentiation states are needed to determine the generalizability of the observed C11orf24-associated phenotypes. Fourth, the biological mechanisms through which C11orf24 regulates stemness and microenvironmental interactions have yet to be fully elucidated. Future studies employing lineage tracing, organoid systems, and genetically engineered models will be necessary to define its downstream regulatory networks. In addition, although re-expression of C11orf24 in C11orf24-silenced T24 cells partially restored SOX2, ALDH1A1, and CD44 expression—supporting the specificity of the knockdown phenotype at the molecular level—functional rescue of malignant phenotypes, rescue of downstream pathways, and in vivo rescue experiments were not performed and will be required to establish mechanistic dependence on C11orf24. Fifth, while the image-based prognostic model demonstrated encouraging performance, prospective multicenter validation will be required before clinical implementation. In addition, the stemness classification was based primarily on CytoTRACE. Although the quartile-based framework was used to preserve the continuous nature of the score, we did not perform independent validation using CytoTRACE2, StemID, or another stemness metric in the present study. Future studies incorporating orthogonal stemness algorithms will be useful for evaluating the robustness of the HStem/DTStem/LStem framework. Finally, the therapeutic compounds identified through pharmacogenomic prediction analyses remain hypothesis-generating and warrant experimental testing in preclinical models.

In summary, our study identifies a highly stem-like epithelial population associated with tumor progression and immune remodeling in BLCA. By integrating single-cell and spatial transcriptomics with computational pathology, we establish a clinically relevant framework connecting epithelial stemness, microenvironmental interactions, and patient outcomes. Furthermore, we identify C11orf24 as a candidate regulator of the stem-like state and a potential therapeutic target. These findings advance our understanding of BLCA biology and provide new opportunities for stemness-directed precision oncology.

## Data availability statement

All data analyzed in this study are publicly available. The bladder cancer (BLCA) single-cell RNA sequencing (scRNA-seq) dataset GSE222315 and the spatial transcriptomics datasets GSE246011, GSE238145, and GSE285715 were obtained from the Gene Expression Omnibus (GEO) database. Bulk RNA-sequencing data for the TCGA-BLCA cohort were downloaded from the UCSC Xena platform. The independent validation cohort GSE31684 was also retrieved from GEO. Any additional data supporting the conclusions of this study are available from the corresponding author upon reasonable request.

## Ethics statement

N/A.

## Funding

This work supported by the National Natural Science Foundation of China (grant number: 82,172,564, 82,303,032, 82,573,338).

## Generative AI statement

Generative AI was used solely for language polishing and grammatical refinement during manuscript preparation. The tool was not used for study design, data generation, data analysis, scientific interpretation, or formulation of the conclusions. The authors reviewed and approved all final manuscript content and remain fully responsible for its accuracy and integrity.

## CRediT authorship contribution statement

**Yingjian Wang:** Formal analysis, Data curation, Conceptualization. **Songxin Zhang:** Data curation, Conceptualization. **Xi Luo:** Formal analysis, Data curation, Conceptualization. **Zhe Wang:** Formal analysis, Data curation, Conceptualization. **Yongjie Wang:** Data curation, Conceptualization. **Ningyang Li:** Formal analysis, Data curation, Conceptualization. **Yu Liu:** Formal analysis, Data curation, Conceptualization. **Jingwei Gao:** Formal analysis, Data curation, Conceptualization. **Ranran Zhang:** Formal analysis, Data curation. **Zhankui Jia:** Formal analysis, Data curation, Conceptualization. **Run Shi:** Formal analysis, Data curation, Conceptualization. **Long Zhang:** Formal analysis, Data curation, Conceptualization.

## Declaration of competing interest

The authors declare that the research was conducted in the absence of any commercial or financial relationships that could be construed as a potential conflict of interest.
