# Supplementary material for: Integrative single-cell and computational pathology analyses reveal a C11orf24-associated stem-like epithelial population linked to tumor microenvironment remodeling and immune evasion in bladder cancer
Source: Transl Oncol. 2026 Oct 21;74:103082. doi: 10.1016/j.tranon.2026.103082 (PMC13718802; doi:10.1016/j.tranon.2026.103082)
Supplement: Supplementary file 1 [file mmc1.docx]

**Supplementary Information**

**Integrative Single-Cell and Computational Pathology Analyses Reveal a C11orf24-Associated Stem-like Epithelial Population that Drives Tumor Microenvironment Remodeling and Immune Evasion in Bladder Cancer**

**Supplementary Data 1 is supplied separately as an Excel workbook and contains Tables S1-S3**

**Supplementary Figure S1. Integration quality assessment and global pathway enrichment across cell types.**

**(A)** UMAP visualization of all cells colored by Seurat clusters.

**(B)** UMAP plots colored by cell type and faceted according to tissue source.

**(C)** Sample composition (left), total cell counts (middle), and sequencing depth (right) across cell types, demonstrating balanced integration and uniform transcriptomic coverage.

**(D)** Bar plot summarizing the number of significantly enriched HALLMARK pathways identified across cell types using seven computational methods (ssGSEA, AUCell, UCell, JASMINE, viper, RRA, and singscore).

**(E)** UpSet plot showing the overlap of activated HALLMARK pathways.

**Supplementary Figure S2. Functional and metabolic characteristics of malignant epithelial cells.**

**(A)** UMAP visualization of malignant epithelial cells colored by Seurat clusters.

**(B)** UMAP visualization colored by tumor stage.

**(C)** Density distribution of CytoTRACE scores.

**(D)** Bar plots showing GSEA enrichment results for HALLMARK, GOBP, and KEGG pathways comparing HStem and LStem cells.

**(E)** Heatmap showing differential metabolic pathway activities between stemness-associated subpopulations based on scMetabolism analysis.

**(F)** Tissue preference analysis of stemness-associated subpopulations across clinical stages using Ro/e scores.

**Supplementary Figure S3. Detailed cell–cell communication analyses among stemness-associated subpopulations**

**(A)** Circular plot showing signaling interactions originating from each cell type.

**(B)** Contributions of individual ligand–receptor pairs to communication between stemness-associated subpopulations and other cell types.

**(C)** Heatmap showing outgoing and incoming interaction strengths and associated signaling pathways.

**(D)** Network centrality analysis of the MIF signaling pathway, illustrating the relative importance of each cell type as sender, receiver, mediator, or influencer.

**(E)** Interaction network of the MIF–(CD74–CXCR4) ligand–receptor pair.

**(F)** Interaction network of the MIF–(CD74–CD44) ligand–receptor pair.

**Supplementary Figure S4. Prognostic modeling and drug sensitivity analyses**

**(A)** Nomogram predicting 1-, 3-, and 5-year overall survival (OS).

**(B)** Dynamic nomogram integrating image-derived risk scores and clinical variables for individualized OS prediction.

**(C)** Calibration curves evaluating predictive accuracy at 1 year (left), 3 years (middle), and 5 years (right).

**(D)** Predicted drug sensitivity comparisons between risk groups for Dasatinib, AZD8055, and Luminespib.

**(E)** Correlation and differential sensitivity analyses of candidate compounds identified from the CTRP and PRISM datasets.

**Supplementary Figure S5. Functional relevance and immune associations of HStem hub genes**

**(A)** Density distributions of six HStem hub genes across all cell types.

**(B)** Density distributions of six HStem hub genes within malignant epithelial cells.

**(C)** Heatmap showing associations between hub genes and immune infiltration features estimated using ESTIMATE, MCP-counter, and ssGSEA algorithms.

**(D)** HALLMARK pathway enrichment heatmap of the six hub genes (C11orf24, CANX, VEGFB, ARF6, ALYREF, and EIF3A).

**(E)** Scatter plots showing correlations between C11orf24 expression and 14 CancerSEA functional states.

**Supplementary Figure S6. Drug sensitivity analysis targeting C11orf24⁺ epithelial cells**

**(A)** Workflow for drug screening targeting C11orf24⁺ epithelial cells.

**(B)** BeyondCell-based quadrant plot illustrating differential drug sensitivity and resistance patterns in C11orf24⁺ epithelial cells.

**(C)** Histograms showing BeyondCell scores of CEPHAELINE and SPIRONOLACTONE in C11orf24-positive and C11orf24-negative epithelial cells.

**(D)** UMAP feature plots displaying single-cell distributions of CEPHAELINE and SPIRONOLACTONE BeyondCell scores, with color gradients representing predicted drug responses.

**(E)** Violin plots comparing BeyondCell scores of CEPHAELINE and SPIRONOLACTONE between HStem and LStem cell populations.
